# Advanced Trajectory Planning and Control for Autonomous Vehicles with Quintic Polynomials

**DOI:** 10.3390/s24247928

**Published:** 2024-12-11

**Authors:** Ma Jin, Mingcheng Qu, Qingyang Gao, Zhuo Huang, Tonghua Su, Zhongchao Liang

**Affiliations:** 1 Faculty of Computing, Harbin Institute of Technology, Harbin 150040, China; jinma@hit.edu.cn (M.J.); thsu@hit.edu.cn (T.S.); 2 Chongqing Research Institute of HIT, Chongqing 401135, China; 3 School of Mechanical Engineering and Automation, Northeastern University, Shenyang 110819, China; gaoqingyang@stumail.neu.edu.cn (Q.G.); 2070142@stu.neu.edu.cn (Z.H.); liangzc@me.neu.edu.cn (Z.L.)

**Keywords:** autonomous vehicle, trajectory design, nonlinear dynamics, fuzzy PID

## Abstract

This paper focuses on the design of vehicle trajectories and their control systems. A method based on quintic polynomials is utilized to develop trajectories for intelligent vehicles, ensuring the smooth continuity of the trajectory and related state curves under varying conditions. The construction of lateral and longitudinal controllers is discussed, which includes a tracking error model derived from the two-degree-of-freedom dynamic model of a two-wheeled vehicle and the application of the Frenet coordinate system transformation. The vehicle tracking performance is regulated by these controllers. Experimental verification on a small intelligent vehicle platform operating on the Ackermann steering principle was conducted. The results confirm the tracking performance of the controllers under different conditions and validate the effectiveness and feasibility of the overall framework of the study.

## 1. Introduction

With the widespread adoption of the concept of intelligent driving and the increasing demand for vehicle-assisted driving, the field of autonomous vehicles has gained significant attention in recent years. Research and popularization of autonomous vehicles have a positive impact on traffic safety, transportation efficiency, and social and economic benefits in modern society. Autonomous vehicles provide substantial convenience for the elderly and disabled, enhancing their quality of life through increased accessibility and inclusivity. Research on autonomous vehicles primarily focuses on perception and environmental understanding [1], localization and map construction [2], and planning and decision-making [3]. The core logic behind the realization of autonomous driving is the vehicle’s ability to track a predetermined trajectory. The key challenges in achieving trajectory tracking are trajectory planning and the design of the vehicle’s controller [4].

To address the trajectory planning problem, this paper employs a quintic polynomial method to generate vehicle trajectories and facilitate lane-change maneuvers. Commonly used lane-change trajectories include sine–cosine function, circular arc, spiral, polynomial function, and clothoid trajectory, etc. Sine–cosine function has the advantage of describing periodic paths [5]; however, they are limited by their singular form and exhibit insufficient accuracy and numerical stability when handling complex trajectories [6]. Circular arc trajectories ensure smooth transitions but lack flexibility and may have discontinuities or abrupt changes at the arc transition points [7]. Spiral trajectories are more flexible than circular arcs but require significant space for smooth transitions, making them unsuitable for sharp turns or sudden directional changes [8]. Clothoid trajectories ensure continuity and smoothness; however, parameter selection is challenging, and high control precision is required [9]. Compared to these existing approaches, quintic polynomial lane-change trajectories offer higher-order continuity, reducing vibrations and shocks [10]. They provide greater accuracy and the ability to describe complex trajectories. This approach optimizes path length, transit time, and energy consumption while ensuring passenger comfort and safety. Due to the continuous derivatives of quintic polynomials, the generated motion trajectories are exceptionally smooth, maintaining throughout, maintaining continuous curvature throughout the vehicle’s movement [11].

After planning an accurate and smooth lane-changing trajectory using a quintic polynomial method, designing a controller to enable the vehicle to track the planned trajectory is essential. In current research, main trajectory tracking controllers for autonomous vehicles include Model Predictive Control (MPC), Sliding Mode Control (SMC), feedforward control, gain scheduling control, and PID control. MPC excels in optimization and constraint handling but has high computational complexity [12]; SMC is effective in dealing with uncertainties and disturbances but suffers from chattering issues [13,14]; feedforward controllers can respond quickly but depend on model accuracy [15]; gain scheduling and adaptive controllers perform well in complex and dynamic environments but are relatively complicated to design and implement [16]. The PID controller is widely used due to its simplicity and robustness [17]. It effectively handles system disturbances and model errors. Despite its advantages, the PID controllers still require precise tuning of the three parameters to achieve optimal control performance [18,19]. Therefore, developing a PID controller that can automatically adjust its parameters is crucial.

To optimize the PID control method, currently proposed approaches include model-based approaches and automatic tuning algorithms. Model-based methods encompass linear quadratic regulation (LQR) and internal model control [20,21,22]. However, model-based approaches require an accurate system model, leading to high computational complexity and limited real-time performance [23]. Automatic tuning algorithms, such as genetic algorithms, particle swarm optimization, and involve iterative computations that consume computational resources [24,25,26]. To reduce computational burden and optimize control structures, employing fuzzy methods to optimize PID controllers holds practical significance. Fuzzy PID controllers integrate fuzzy logic with traditional PID control, effectively handling nonlinear and uncertain systems while enhancing control system robustness and adaptability. Even without precise mathematical models, fuzzy PID controllers achieve excellent control performance and smooth transitions through fuzzy rules and adaptive adjustments.

Based on the above information to achieve the desired trajectory tracking effect, this paper employs the quintic polynomial method to design an ideal trajectory and adopts a fuzzy PID controller to ensure the vehicle follows the ideal trajectory. The main contributions are as follows:

- To achieve a smooth trajectory transition and ensure occupant comfort and safety, an ideal trajectory is designed using the quintic polynomial method;
- A fuzzy PID controller is designed to overcome the tuning challenges and enhance the robustness of the controller;
- The superiority and effectiveness of the proposed method are validated through testing on an intelligent experimental vehicle.

The article is divided into three parts. The first part includes vehicle model establishment, quintic polynomial trajectory design, and trajectory tracking error model design. The second part focuses on the design of the fuzzy PID controller. The third part presents experimental validation, which verifies the effectiveness of the vehicle controllers under two different conditions using an intelligent driven-motors small car with Ackermann front-wheel steering.

## 2. Model Establishment and Trajectory Generation

### 2.1. Vehicle Dynamics Model

Vehicle dynamics models are mathematical representations based on physical principles. They are commonly categorized by degrees of freedom into two-degree-of-freedom (2-DOF) models, three-degree-of-freedom (3-DOF) models, four-degree-of-freedom (4-DOF) models, five-degree-of-freedom (5-DOF) models, and seven-degree-of-freedom (7-DOF) models. The 2-DOF model is a simplified model that considers the vehicle as a single rigid body with two degrees of freedom: lateral movement along the *y*-axis and rotation around the *z*-axis. This model is commonly used for vehicle handling analysis, especially in evaluating overall stability, steering response, and cornering behavior [27,28].

**Figure 1:**
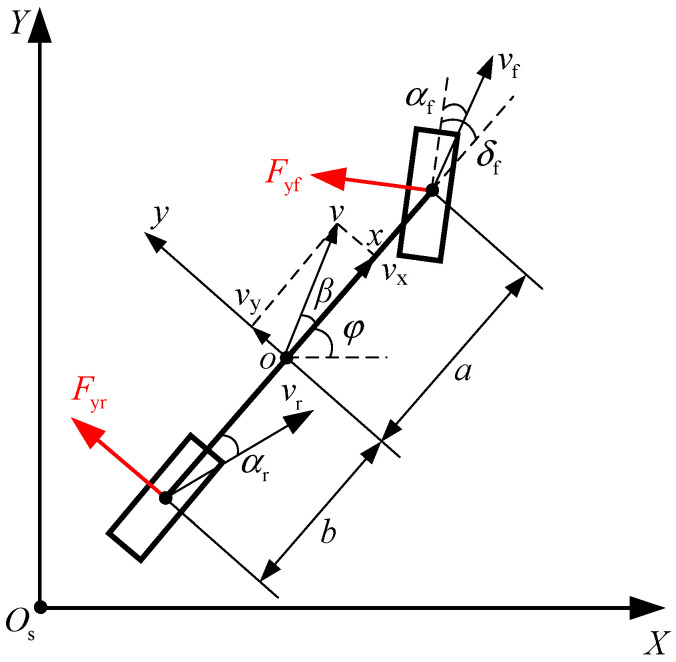
Two-Degree-of-Freedom Dynamic Model of a Two-Wheel Vehicle.

The 2-DOF model offers a balanced trade-off between accuracy and computational efficiency, making it suitable for real-time simulation and control applications. In contrast, higher degrees of freedom require more complex equations and additional parameters, increasing computational costs and complicating model establishment, which makes them less suitable for real-time applications. Therefore, based on the research content of this chapter, a 2-DOF vehicle dynamic model is sufficient for basic vehicle handling analysis and control. Its schematic diagram is shown in Figure 1 [29,30].

In the 2-DOF dynamic model of a two-wheeled vehicle, the front wheel is the steering wheel, while the rear wheel does not steer, meaning $\delta_{r}=0$, and is generally aligned with the vehicle’s body axis. A vehicle-fixed coordinate system *xoy* is established at the center of mass. The velocity at the center of mass is *v*, and the vehicles sideslip angle of is *β*. The front axle length is *a*, the front wheel speed is *v*_f_, the front wheel sideslip angle is *α*_f_, and the lateral force experienced is *F_y_*_f_. The rear axle length is *b*, the rear wheel speed is *v*_r_, the rear wheel sideslip angle is *α*_r_, and the lateral force experienced is *F*_yr_.

The sideslip angles of the front and rear wheels are opposite to the direction of lateral forces. Neglecting motion along the longitudinal x-axis, based on the vehicle’s lateral translation on the y-axis and rotation about the z-axis, the following relationships can be established:

(1) $$\left\{\begin{array}{l} ma_{y}=F_{\mathrm{yf}}\cos\delta_{f}+F_{\mathrm{yr}}\\ F_{\mathrm{yf}}\left(\cos\delta_{f}\right)a-F_{\mathrm{yr}}b=I\ddot{\varphi } \end{array}\right.$$

where *m* is the mass of the entire vehicle (including suspension, tires, etc.), *a*_y_ is the lateral acceleration, *I* is the moment of inertia about the z-axis, and $\ddot{\varphi }$ is the yaw rate of the vehicle. Assuming that the steering angle of the front wheel is small during vehicle motion, i.e., $\cos\delta_{f}\approx 1$, we can simplify Equation (1) as follows:

(2) $$\left\{\begin{array}{l} ma_{y}=C_{\alpha f}\alpha_{f}+C_{\alpha r}\alpha_{r}\\ I\ddot{\varphi }=aC_{\alpha f}\alpha_{f}-bC_{\alpha r}\alpha_{r} \end{array}\right.$$

where $C_{\alpha f}$ and $C_{\alpha r}$ represent the front and rear tire cornering stiffness, respectively. Combining Equations (1) and (2), the vehicle dynamics model is given by:

(3) $$\left\{\begin{array}{l} m\left({\dot{v}}_{y}+v_{x}\dot{\varphi }\right)=C_{\alpha f}\left(\frac{a\dot{\varphi }+v_{y}}{v_{x}}-\delta_{f}\right)+C_{\alpha r}\left(\frac{v_{y}-b\dot{\varphi }}{v_{x}}\right)\\ I\ddot{\varphi }=aC_{\alpha f}\left(\frac{a\dot{\varphi }+v_{y}}{v_{x}}-\delta_{f}\right)-bC_{\alpha r}\left(\frac{v_{y}-b\dot{\varphi }}{v_{x}}\right) \end{array}\right.$$

where ${\dot{v}}_{y}=\dot{y}$ and (3) can be simplified and written in the form of state-space equations as:

(4) $$\left[\begin{array}{c} \ddot{y}\\ \ddot{\varphi } \end{array}\right]=\left[\begin{array}{cc} \frac{C_{\alpha f}+C_{\alpha r}}{mv_{x}} & \frac{aC_{\alpha f}-bC_{\alpha r}}{mv_{x}}\\ \frac{aC_{\alpha f}-bC_{\alpha r}}{Iv_{x}} & \frac{a^{2}C_{\alpha f}+b^{2}C_{\alpha r}}{Iv_{x}} \end{array}\right]\left[\begin{array}{c} \dot{y}\\ \dot{\varphi } \end{array}\right]+\left[\begin{array}{c} -\frac{C_{\alpha f}}{m}\\ -\frac{aC_{\alpha f}}{I} \end{array}\right]\delta_{f}$$

### 2.2. Vehicle Trajectory Planning Based on Quintic Polynomial Method

In the design of vehicle tracking trajectories, two crucial metrics are safety and comfort. Commonly used lane-changing trajectories include sinusoidal, circular arc, spiral, polynomial function, and loop curve trajectories [1,2,3]. Sinusoidal lane-changing trajectories are based on sine or cosine functions to design trajectories from the current lane to the target lane. However, a major drawback is that the maximum curvature occurs at the start and end points, resulting in the highest lateral acceleration at these points, which does not satisfy the requirement of zero curvature at the start and end points of the lane-changing trajectory. Circular arc lane-change trajectories consist of two circular arcs at the start and end points of the lane change, connected by a straight lane segment. This discontinuity in the trajectory curve at the endpoints of the circular arcs causes sudden changes in lateral acceleration, significantly increasing the difficulty of control. Spiral lane-change trajectories involve continuous turning over a long distance, forming a spiral curve suitable for gradual deceleration or acceleration.

The polynomial function lane-change trajectory effectively avoids the drawbacks of the aforementioned trajectories by providing smooth transitions and low lateral acceleration without sudden changes, achieved through higher-order polynomial functions. The most typical examples are the third-order and the fifth-order polynomial lane-change trajectory. The main difference between the two lies in continuity: the third-order polynomial trajectory ensures continuous displacement and velocity, but not acceleration, whereas the quintic polynomial provides continuous displacement, velocity, and acceleration, thereby enhancing trajectory smoothness. Although this study focuses on uniform-speed lane-changing, designing acceleration is necessary for trajectory tracking during deceleration at intersections to ensure smooth and continuous acceleration curves. Fitting the tracking trajectory with a quintic polynomial can achieve better operational results.

**Figure 2:**
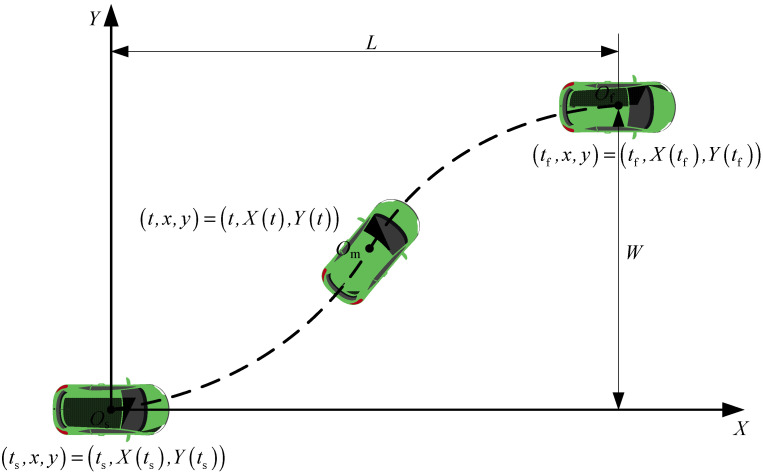
Schematic Diagram of a General Fifth-order Polynomial Trajectory.

The general form of a quintic polynomial trajectory is depicted in Figure 2, with a reference coordinate system *XO*_s_*Y* established at the initial position of the autonomous vehicle. Here, the starting point of the trajectory is denoted as the coordinate origin *O*_s_ (the center of the autonomous vehicle), *O*_f_ represents the endpoint of the trajectory, and *O*_m_ represents a certain position of the autonomous vehicle at time *t* during its travel. The *X*-axis corresponds to the direction of the lane, and the *Y*-axis is perpendicular to the lane. *L* represents the longitudinal length of the trajectory, while *W* represents the lateral length of the trajectory.

As shown in Figure 2, at the initial moment *t*_s_, the lateral and longitudinal displacements, velocities, and accelerations of the autonomous vehicle are denoted as $X\left(t_{s}\right)$, $\dot{X}\left(t_{s}\right)$, $\ddot{X}\left(t_{s}\right)$, $Y\left(t_{s}\right)$, $\dot{Y}\left(t_{s}\right)$, and $\ddot{Y}\left(t_{s}\right)$, respectively. At the termination moment *t*_f_, the lateral and longitudinal displacements, velocities, and accelerations are denoted as $X\left(t_{f}\right)$, $\dot{X}\left(t_{f}\right)$, $\ddot{X}\left(t_{f}\right)$, $Y\left(t_{f}\right)$, $\dot{Y}\left(t_{f}\right)$, and $\ddot{Y}\left(t_{f}\right)$, respectively. The total time spent on the entire trajectory is denoted as *T*.

(5) $$T=t_{f}-t_{s}$$

As is well known, unlike robots, cars cannot perform lateral motion independently; lateral motion is induced by longitudinal movement. The designed trajectory is subject to constraints such as tangent, curvature, velocity, and acceleration. Therefore, at a certain moment *t* during the autonomous vehicle’s travel, the lateral and longitudinal state variables are denoted as $X\left(t\right)$, $\dot{X}\left(t\right)$, $\ddot{X}\left(t\right)$, $Y\left(X\right)$, $\dot{Y}\left(X\right)$, and $\ddot{Y}\left(X\right)$. However, in the subsequent control system design, the input state variables need to be a function of time *t*. Consequently, the lateral state variables with respect to *X* need to be transformed. The motion equation for lateral displacement $Y\left(t\right)$ with respect to time *t* is:

(6) $$Y\left(t\right)=Y\left(X\right)=Y\left(X\left(t\right)\right)$$

Derived from Equation (6), the relationship between lateral velocity $\dot{Y}\left(t\right)$ and time is obtained as:

(7) $$\dot{Y}\left(t\right)=\frac{dy}{dx}\cdot \frac{dx}{dt}=\dot{Y}\left(X\right)\cdot \dot{X}\left(t\right)$$

Derived from Equation (7), the relationship between lateral velocity $\dot{Y}\left(t\right)$ and time is obtained as:

(8) $$\begin{array}{l} \ddot{Y}\left(t\right)=\frac{d}{dt}\left(\frac{dy}{dx}\cdot \frac{dx}{dt}\right)=\frac{d\left(\frac{dy}{dx}\right)}{dt}\cdot \frac{dx}{dt}+\frac{dy}{dx}\cdot \frac{d^{2}x}{dt^{2}}\\ \;\;\;\;\;\;=\ddot{Y}\left(X\right)\cdot \dot{X}{\left(t\right)}^{2}+\dot{Y}\left(X\right)\cdot \ddot{X}\left(t\right) \end{array}$$

The quintic polynomial trajectory function can be represented as follows:

(9) $$\left\{\begin{array}{l} X\left(t\right)=\sum_{i=0}^{5}a_{i}\times t^{i}\\ Y\left(X\right)=\sum_{i=0}^{5}b_{i}\times X^{i} \end{array}\right.$$

where the coefficients of the designed quintic polynomial are:

(10) $$\left\{\begin{array}{l} a=\left[\begin{array}{cccccc} a_{0} & a_{1} & a_{2} & a_{3} & a_{4} & a_{5} \end{array}\right]\\ b=\left[\begin{array}{cccccc} b_{0} & b_{1} & b_{2} & b_{3} & b_{4} & b_{5} \end{array}\right] \end{array}\right.$$

By combining the three initial state variables, three terminal state variables of the autonomous vehicle trajectory, and Equations (5)–(8), the lateral and longitudinal state variables X, $\dot{X}$, $\ddot{X}$, Y, $\dot{Y}$, and $\ddot{Y}$ at a certain moment *t* during the autonomous vehicle’s travel within the time interval *T* can be calculated as follows:

(11) $$\left\{\begin{array}{l} X=a_{0}+a_{1}t+a_{2}t^{2}+a_{3}t^{3}+a_{4}t^{4}+a_{5}t^{5}\\ \dot{X}=a_{1}+2\cdot a_{2}t+3\cdot a_{3}t^{2}+4\cdot a_{4}t^{3}+5\cdot a_{5}t^{4}\\ \ddot{X}=2\cdot a_{2}+6\cdot a_{3}t+12\cdot a_{4}t^{2}+20\cdot a_{5}t^{3} \end{array}\right.$$

(12) $$\left\{\begin{array}{l} Y=b_{0}+b_{1}X+b_{2}X^{2}+b_{3}X^{3}+b_{4}X^{4}+b_{5}X^{5}\\ \dot{Y}=\dot{Y}\left(X\right)\cdot \dot{X}\\ \ddot{Y}=\ddot{Y}\left(X\right)\cdot {\dot{X}}^{2}+\dot{Y}\left(X\right)\cdot \ddot{X} \end{array}\right.$$

where the coefficients in (10) and $\dot{Y}\left(X\right)$, and $\ddot{Y}\left(X\right)$ are given as follows:

(13) $$\left\{\begin{array}{l} \dot{Y}\left(X\right)=b_{1}+2\cdot b_{2}X+3\cdot b_{3}X^{2}+4\cdot b_{4}X^{3}+5\cdot b_{5}X^{4}\\ \ddot{Y}\left(X\right)=2\cdot b_{2}+6\cdot b_{3}X+12\cdot b_{4}X^{2}+20\cdot b_{5}X^{3} \end{array}\right.$$

(14) $$\begin{array}{l} \left\{\begin{array}{c} a_{0}=X\left(t_{s}\right)\\ a_{1}=\dot{X}\left(t_{s}\right)\\ a_{2}=\frac{\ddot{X}\left(t_{s}\right)}{2} \end{array}\right.\;\left[\begin{array}{c} a_{3}\\ a_{4}\\ a_{5} \end{array}\right]=A_{1}^{-1}\cdot B_{1}\\ \left\{\begin{array}{c} b_{0}=Y\left(t_{s}\right)\\ b_{1}=\dot{Y}\left(t_{s}\right)\\ b_{2}=\frac{\ddot{Y}\left(t_{s}\right)}{2} \end{array}\right.\;\left[\begin{array}{c} b_{3}\\ b_{4}\\ b_{5} \end{array}\right]=A_{2}^{-1}\cdot B_{2} \end{array}$$

where matrices $A_{1}$, $B_{1}$, $A_{2}$, and $B_{2}$ are computed as follows:

(15) $$A_{1}=\left[\begin{array}{ccc} T^{3} & T^{4} & T^{5}\\ 3\times T^{2} & 4\times T^{3} & 5\times T^{4}\\ 6\times T & 12\times T^{2} & 20\times T^{3} \end{array}\right]$$

(16) $$B_{1}=\left[\begin{array}{c} X\left(t_{f}\right)-a_{0}-a_{1}\cdot T-a_{2}\cdot T^{2}\\ \dot{X}\left(t_{f}\right)-a_{1}-2\times a_{2}\cdot T\\ \ddot{X}\left(t_{f}\right)-2\times a_{2} \end{array}\right]$$

(17) $$A_{2}=\left[\begin{array}{ccc} L^{3} & L^{4} & L^{5}\\ 3\times L^{2} & 4\times L^{3} & 5\times L^{4}\\ 6\times L & 12\times L^{2} & 20\times L^{3} \end{array}\right]$$

(18) $$B_{2}=\left[\begin{array}{c} Y\left(t_{f}\right)-b_{0}-b_{1}\cdot L-b_{2}\cdot L^{2}\\ \dot{Y}\left(t_{f}\right)-b_{1}-2\times b_{2}\cdot L\\ \ddot{Y}\left(t_{f}\right)-2\times b_{2} \end{array}\right]$$

At the same time, we can calculate the angle *theta* between the tangent direction of the trajectory and the X-axis, as well as the curvature *k* of the trajectory:

(19) $$\left\{\begin{array}{l} theta=\arctan\left(\dot{Y}\left(X\right)\right)\\ k=\frac{\ddot{Y}\left(X\right)}{{\left[1+{\left(\dot{Y}\left(X\right)\right)}^{2}\right]}^{1.5}} \end{array}\right.$$

### 2.3. Tracking Error Model Based on Frenet Coordinate System

**Figure 3:**
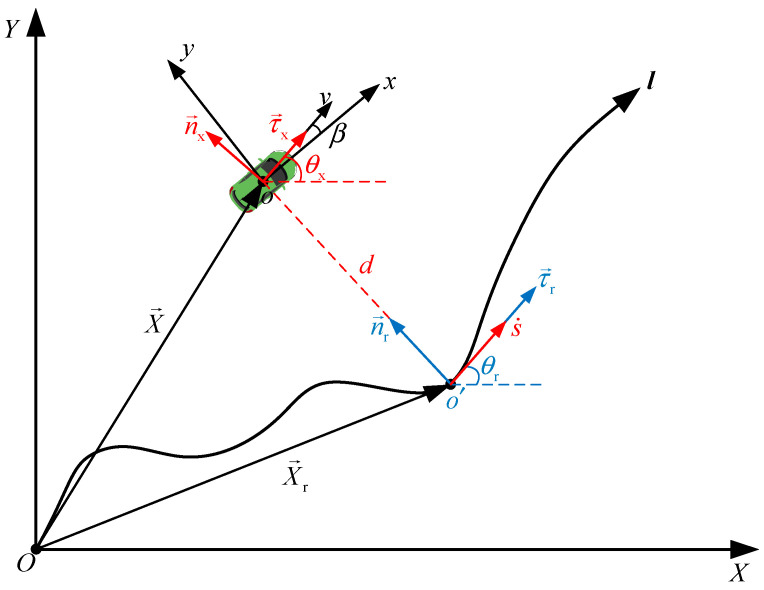
Schematic Diagram of Trajectory Tracking.

As shown in Figure 3, the current sideslip angle at the center of mass (the center of mass is denoted as point *o*) is *β*, with a velocity magnitude of *v* and a heading angle of $\theta_{x}$. In the Frenet coordinate system, the current velocity direction of the intelligent vehicle aligns with the tangent vector ${\vec{\tau }}_{x}$, and the normal direction with the normal vector ${\vec{n}}_{x}$. The tracking trajectory is *l* and the projection point of *o* on *l* is point $\overset{\prime }{o}$. The projected tangent vector at $\overset{\prime }{o}$ is ${\vec{\tau }}_{r}$ and the normal vector is ${\vec{n}}_{r}$. The distance from the point $\overset{\prime }{o}$ to point *o* represents the lateral error *d*. The longitudinal cumulative arc length from the starting point of the reference trajectory to the projection point $\overset{\prime }{o}$ is *s*. The projected velocity magnitude is $\dot{s}$ and the heading angle is $\theta_{r}$. Therefore, the heading error is $e_{\theta }=\theta_{x}-\theta_{r}$.

From point *O*, vectors $\vec{X}$ and ${\vec{X}}_{r}$ are drawn to points *o* and $\overset{\prime }{o}$, respectively. When $\vec{X}$ is the actual position vector of the intelligent vehicle and ${\vec{X}}_{r}$ is the projected position vector. In the triangle *Oo*$\overset{\prime }{o}$ we can derive:

(20) $$\left\{\begin{array}{l} \dot{\vec{X}}=v{\vec{\tau }}_{x}\\ {\dot{\vec{X}}}_{r}=\dot{s}\vec{\tau }\\ {\vec{X}}_{r}+d{\vec{n}}_{r}=\vec{X} \end{array}\right.$$

From Equation (20), taking the derivative of *d* yields:

(21) $$\left\{\begin{array}{l} \frac{d{\vec{n}}_{r}}{dt}=\frac{d{\vec{n}}_{r}}{ds}\cdot \frac{ds}{dt}\\ d=\left(v{\vec{\tau }}_{x}-\dot{s}{\vec{\tau }}_{r}\right)\cdot {\vec{n}}_{r}+\left(\vec{X}-{\vec{X}}_{r}\right)\cdot \frac{d{\vec{n}}_{r}}{dt} \end{array}\right.$$

From the Frenet formulas for a two-dimensional curve, we obtain:

(22) $$\left\{\begin{array}{c} \frac{d{\vec{\tau }}_{x}}{ds}=k{\vec{n}}_{x}\\ \frac{d{\vec{n}}_{x}}{ds}=-k{\vec{\tau }}_{x} \end{array}\right.$$

where *k* represents the curvature of the reference trajectory curve. From Equations (21) and (22), we obtain:

(23) $$\dot{d}=v\sin\left(\theta_{x}-\theta_{r}\right)=v\sin e_{\theta }$$

Combine (21)–(24) and taking the derivative of (20) yields:

(24) $$\dot{s}{\vec{\tau }}_{r}+v\sin e_{\theta }{\vec{n}}_{r}+d\left(-k\dot{s}{\vec{\tau }}_{r}\right)=v{\vec{\tau }}_{x}$$

Multiplying both sides of Equation (24) by ${\vec{\tau }}_{r}$ yields:

(25) $$\dot{s}=\frac{v\cos e_{\theta }}{1-kd}$$

From the relationship among the heading angle θ, the yaw angle φ, and the sideslip angle β, we can further transform Equation (23):

(26) $$\dot{d}=v\sin\left(\varphi_{x}+\beta_{x}-\theta_{r}\right)$$

where *v*_x_ is the longitudinal velocity at the intelligent vehicle’s center of mass, *v*_y_ is the lateral velocity, and $\varphi_{x}-\theta_{r}$ can be regarded as a small quantity. Therefore, Equation (26) can be further simplified as follows:

(27) $$\dot{d}=v_{y}+v_{x}\sin\left(\varphi_{x}-\theta_{r}\right)$$

Meanwhile Equation (25) can be simplified as follows:

(28) $$\dot{s}=\frac{v_{x}-v_{y}\cos\left(\varphi_{x}-\theta_{r}\right)}{1-kd}$$

In summary, in the Frenet coordinate system (see Figure 3), the lateral error is *e*_d_ = *d*, and the error between the yaw angle and the heading angle is $e_{\varphi }=\varphi_{x}-\theta_{r}$, and due to the smoothness of the road, we can ignore the second derivative of $\theta_{r}$, so ${\ddot{\theta }}_{r}=0$. Also, assuming *v*_x_ is constant, the equation is yielded as follows:

(29) $$\left\{\begin{array}{l} v_{y}={\dot{e}}_{d}-v_{x}e_{\varphi }\\ {\dot{v}}_{y}={\ddot{e}}_{d}-v_{x}{\dot{e}}_{\varphi }\\ \dot{\varphi }={\dot{e}}_{\varphi }+{\dot{\theta }}_{r}\\ \ddot{\varphi }={\ddot{e}}_{\varphi }\\ \dot{s}=\frac{v_{x}-v_{y}\cos\left(\varphi_{x}-\theta_{r}\right)}{1-kd} \end{array}\right.$$

Combining Equation (4), we can obtain the state-space equations for the tracking error model in the Frenet coordinate system as follows:

(30) $${\dot{e}}_{\mathrm{rr}}=Ae_{\mathrm{rr}}+Bu+C{\dot{\theta }}_{r}$$

where $u=\delta_{f}$, $e_{\mathrm{rr}}={\left[\begin{array}{cccc} ed & \dot{e}d & e\varphi & \dot{e}\varphi \end{array}\right]}^{T}$

$$A=\left[\begin{array}{cccc} 0 & 1 & 0 & 0\\ 0 & \frac{C_{\alpha f}+C_{\alpha r}}{mv_{x}} & -\frac{C_{\alpha f}+C_{\alpha r}}{m} & \frac{aC_{\alpha f}-bC_{\alpha r}}{mv_{x}}\\ 0 & 0 & 0 & 1\\ 0 & \frac{aC_{\alpha f}-bC_{\alpha r}}{Iv_{x}} & -\frac{aC_{\alpha f}-bC_{\alpha r}}{I} & \frac{a^{2}C_{\alpha f}+b^{2}C_{\alpha r}}{Iv_{x}} \end{array}\right]\;B=\left[\begin{array}{c} 0\\ -\frac{C_{\alpha f}}{m}\\ 0\\ -\frac{aC_{\alpha f}}{I} \end{array}\right]\cdot C=\left[\begin{array}{c} 0\\ \frac{aC_{\alpha f}-bC_{\alpha r}}{mv_{x}}-v_{x}\\ 0\\ \frac{a^{2}C_{\alpha f}+b^{2}C_{\alpha r}}{Iv_{x}} \end{array}\right],$$

where the error state matrix $e_{\mathrm{rr}}$ consists of the lateral position and velocity error *e*_d_ and ${\dot{e}}_{d}$, the error between yaw angle and heading angle $e_{\varphi }$, and the derivative of the heading angle error ${\dot{e}}_{\varphi }$:

(31) $$\left\{\begin{array}{l} e_{d}=-\left(x-x_{d}\right)\sin\theta_{d}+\left(y-y_{d}\right)\cos\theta_{d}\\ {\dot{e}}_{d}=v_{y}\cos\left(\varphi -\theta_{r}\right)+v_{x}\sin\left(\varphi -\theta_{r}\right)\\ e_{\varphi }=\varphi -\theta_{r}\\ {\dot{e}}_{\varphi }=\dot{\varphi }-k_{d}\frac{v_{x}\cos\left(\varphi -\theta_{r}\right)-v_{y}\sin\left(\varphi -\theta_{r}\right)}{1-k_{d}e_{d}} \end{array}\right.$$

similarly, longitudinal position *e*_s_ and velocity errors $e_{v}$ yield as follows;

(32) $$\left\{\begin{array}{l} e_{s}=-\left(x-x_{d}\right)\cos\theta_{d}+\left(y-y_{d}\right)\sin\theta_{d}\\ e_{v}=v_{x}-\dot{s} \end{array}\right.$$

When the error system (30) is stable, the tracking error converges to zero, indicating that the target vehicle enable track to the ideal path.

## 3. Controller Design

### 3.1. Lateral Fuzzy PID Controller Design

Fuzzy PID control is a method that combines fuzzy logic with traditional PID control. It mainly includes three components: the proportional, integral, and derivative components. The proportional component calculates the difference between the current process variable and the reference value, and multiplies this difference by the proportional gain factor. The integral component calculates the accumulated error over time and multiplies the integral gain factor. The derivative component calculates the rate of change in the process variable and multiplies the derivative gain factor.

In a traditional PID control system, the gain factors are fixed and require manual adjustment based on the specific control system. However, in a fuzzy PID control system, the gain factors are dynamic and determined by fuzzy rules. These rules associate the current state of the system with appropriate gain values, allowing real-time updates of the gain factors according to the current system state and the set sampling time.

**Figure 4:**
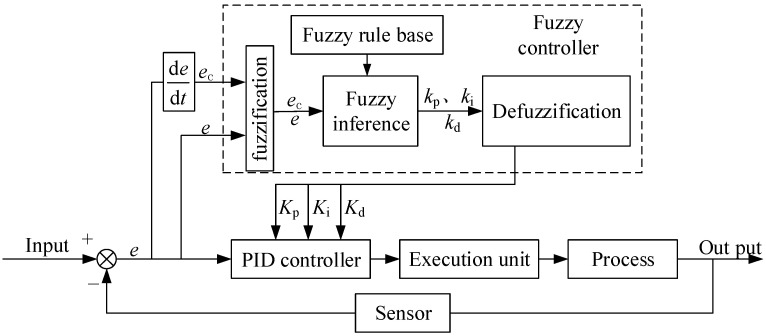
Principle of Fuzzy PID Control.

The gain factors are primarily described by defining fuzzy sets for the input and output variables. These fuzzy sets are represented using linguistic terms, such as “low”, “medium”, and “high”, which are associated with numerical values. Control parameters for the output are then determined through a set of fuzzy rules. The basic principle of the fuzzy PID controller is illustrated in Figure 4.

Using fuzzy PID control to regulate the front-wheel steering angle of an intelligent vehicle during trajectory tracking involves controlling the front-wheel steering angle $\delta_{f}$. The lateral displacement error *e*_y_ and the heading angle error *e*_φ_ are multiplied by different weighting coefficients and then summed to obtain the lateral weighted error *e*_yφ_, as shown in the following equation:

(33) $$e_{y\varphi }=\lambda_{1}e_{y}+\lambda_{2}e_{\varphi }$$

In a fuzzy controller, the input variables typically need to be fuzzified to represent their fuzzy nature. This process usually involves dividing the input variables into multiple fuzzy sets and representing them by a membership function. Similarly, the output variables are also fuzzified so that the results of the fuzzy logic can be mapped back to actual output values. This typically includes mapping the output values to multiple fuzzy sets and assigning a membership function to each set.

In this section, the fuzzy control part of the lateral fuzzy PID controller has two inputs and three outputs. The two inputs are the lateral weighted error *e*_yφ_ and its rate of change *e*_c1_. The three outputs, which after defuzzification, are the three adjustable parameters of the PID controller: *K*_p1_, *K*_i1_, and *K*_d1_. By adjusting these PID control parameters, the real-time stability of the intelligent vehicle’s motion is achieved.

**Figure 5:**
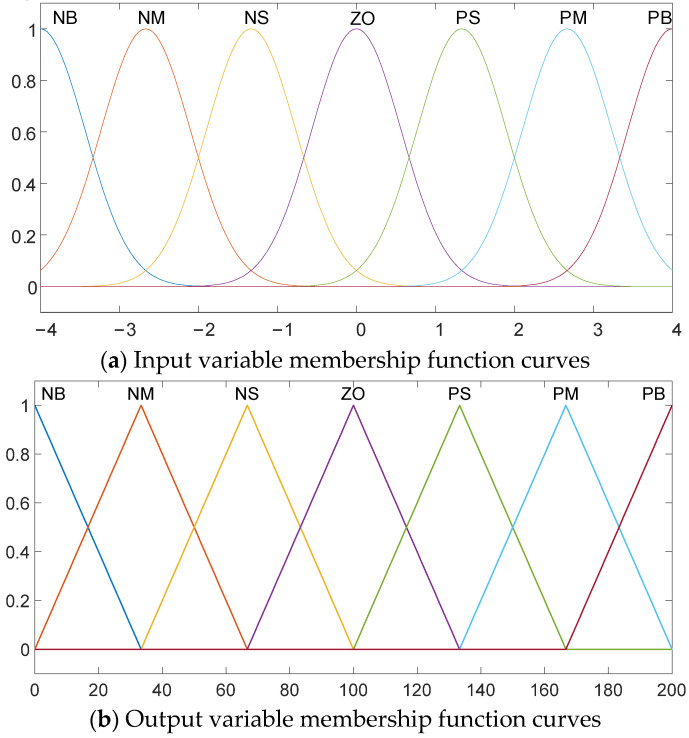
Input and output variable membership function curves.

For implementing the lateral fuzzy control part, it is necessary to define fuzzy sets for the inputs and outputs. Gaussian and triangular membership will be used as the fuzzification functions for the input and output variables. The function curves are shown in Figure 5.

The fuzzy sets for all input and output variables are divided into seven levels: PB, PM, PS, ZO, NS, NM, and NB, corresponding to Positive Big, Positive Medium, Positive Small, Zero, Negative Small, Negative Medium, and Negative Big, respectively. During the fuzzification process of the input and output variables, the degree of membership for each variable in each fuzzy set is calculated based on the membership functions.

The domain of the fuzzy control inputs and outputs is determined by the requirements of the control system, the range of values that the fuzzy sets can represent, the rule base, and performance standards. In order to meet the precision and stability requirements of lateral control algorithms for intelligent vehicles with time-varying nonlinear systems, as well as the subsequent simulation stage’s needs for vehicle characteristics and debugging, the domains of the input variable *e*_yφ_ and *e*_c1_ are both set to [−4, 4], while the domains of the output variables *K*_p1_, *K*_i1_, and *K*_d1_ are set to [0, 200], [0, 1], and [0, 1].

In the lateral fuzzy PID control, the fuzzy controller takes the weighted error between the heading angle and lateral position, along with the rate of change in error *e*_c1_, as inputs, and outputs three parameters of the PID controller: *K*_p1_, *K*_i1_, and *K*_d1_, as shown in Equation (33). The intelligent vehicle minimizes the lateral displacement error *e*_*y*_ and heading angle error approaching zero by adjusting the front wheel steering angle. At various stages of lateral control, the construction of the fuzzy table enables real-time adjustment of the three tunable parameters in the PID controller, thereby impacting the stability of the intelligent vehicle’s lateral movement.

Firstly, the selection of the *K*_p1_ is critical in the operation of the lateral controller. On one hand, as the lateral weighted error *e*_yφ_ gradually decreases, a larger *K*_p1_ value is required to quickly bring the intelligent vehicle closer to the reference trajectory, raising the system’s response and enhancing control accuracy. However, an excessively large *K*_p1_ can cause the system to oscillate, so the selection of *K*_p1_ needs to balance the controller’s response speed and stability. On the other hand, when the intelligent vehicle is moving at a higher longitudinal speed, a smaller *K*_p1_ value is preferred to quickly eliminate lateral deviation between the actual position and the reference trajectory. A smaller *K*_p1_ value results in smaller output variations, reducing the interference with the lateral control and helping to eliminate lateral error more swiftly. Moreover, when the lateral weighted error is large, it is necessary to appropriately reduce the *K*_p1_ value to prevent the output from exceeding the adjustment range of the lateral controller, thereby ensuring effective control.

Secondly, the selection of the *K*_i1_ is important. Proper adjustment of the *K*_i1_ value can reduce the steady-state error accumulated during the *K*_p1_ regulation process. However, when the intelligent vehicle’s longitudinal speed is high and the lateral weighted error is significant, it is necessary to appropriately lower the *K*_i1_ in coordination with *K*_d1_ adjustment. This helps to prevent system oscillations and minimizes the influence of the lateral controller on the front wheel steering angle of the intelligent vehicle, thereby maintaining system stability.

Thirdly, the selection of the *K*_d1_ is crucial. When the lateral control system of the intelligent vehicle requires a larger *K*_p1_, it is essential to select an appropriate *K*_d1_ value in conjunction with *K*_i1_ to prevent significant oscillations in the heading angle and lateral position error. The specific value of *K*_d1_ should be chosen based on the degree of lateral weighted error fluctuation: the greater the fluctuation, the larger the *K*_d1_ value, and vice versa. This approach helps to dampen oscillations and stabilize the system’s response. Combining the above analysis and adjustments from multiple simulations and experiments in subsequent chapters, the Fuzzy Rules in the lateral fuzzy PID controller are shown in Table 1. Input the above rules into the MATLAB Fuzzy Control Toolbox and conduct dynamic simulation.

**Figure 6:**
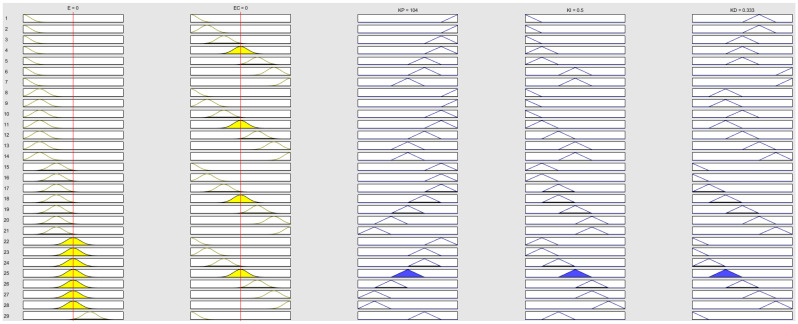
Lateral dynamic simulation results.

**Figure 7:**
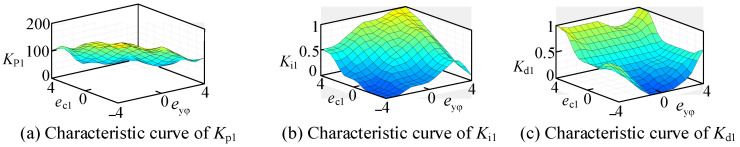
Lateral characteristic curves of input and output.

The results are shown in Figure 6. The characteristic curves of the input and output variables are presented in Figure 7.

To obtain accurate output values, it is necessary to defuzzify the fuzzy output values of the fuzzy controller. In this paper, the centroid method is used for defuzzification. This method provides smooth control characteristics and can respond sensitively to small changes. The calculation expression for centroid defuzzification is as follows:

(34) $$M=\sum_{k=1}^{n}m_{k}u_{c}\left(m_{k}\right)/\sum_{k=1}^{n}u_{c}\left(m_{k}\right)$$

where *n* is the number of output quantization levels, *M* is the precise output value, *m_k_* is the value within the domain of the fuzzy control quantity, and *u*_c_(*m_k_*) is the membership value of *m_k_*.

### 3.2. Vertical Fuzzy PID Controller Design

The longitudinal weighted error *e*_xv_ is calculated by multiplying the longitudinal displacement error *e*_x_ and the velocity error *e*_v_ by different weighting coefficients, then summing them, as shown in the following equation:

(35) $$e_{\mathrm{xv}}=\omega_{1}e_{x}+\omega_{2}e_{v}$$

**Figure 8:**
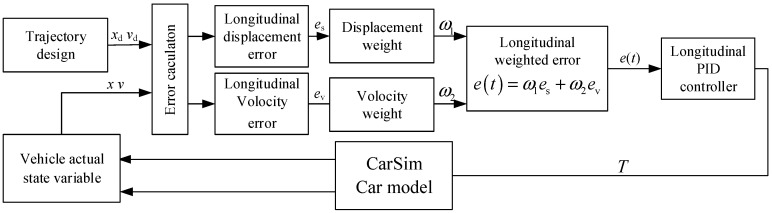
Longitudinal PID Displacement–Velocity Controller Principle.

In the longitudinal control system of the intelligent vehicle, the longitudinal displacement error and velocity error are used to evaluate whether the vehicle’s position and speed have reached the desired values. Based on the different importance of these errors and the stability requirements of the overall longitudinal control. These weight errors are then combined into a weighted error signal, which serves as the input signal of the PID controller to improve the control system performance. The principle is illustrated in Figure 8.

As shown in Figure 8, the longitudinal displacement error and the velocity error are each multiplied by different weight coefficients and then summed. The weighted error signal *e*(*t*) is calculated as follows:

(36) $$e\left(t\right)=\omega_{1}e_{s}+\omega_{2}e_{v}$$

where *e*_s_ and *e*_v_ represent the longitudinal displacement error and velocity error, respectively, while *ω*_1_ and *ω*_2_ are the corresponding weight coefficients. The weighted error signal serves as the input signal to the PID controller. Finally, the output signal from the controller is applied to the actuator unit of the intelligent vehicle’s longitudinal system, forming the longitudinal PID controller as follows:

(37) $$u\left(t\right)=K_{p}e\left(t\right)+K_{i}\int_{0}^{t}e\left(t\right)dt+K_{d}\frac{de\left(t\right)}{dt}$$

In the longitudinal fuzzy PID controller, the fuzzy control component has two inputs and three outputs. The two inputs are the longitudinal weighted error *e*_xv_ of the intelligent vehicle and its rate of change *e*_c2_. The three outputs, after defuzzification, are the adjustable parameters of the PID controller: *K*_p2_, *K*_i2_, and *K*_d2_.

In implementing the longitudinal fuzzy control, Gaussian and triangular membership functions are used as the fuzzification functions for the input and output variables with function curves identical to those in Figure 5, which will not be shown again here.

Similarly, all input and output variables are divided into seven fuzzy sets: PB, PM, PS, ZO, NS, NM, and NB standing for Positive Big, Positive Medium, Positive Small, Zero, Negative Small, Negative Medium, and Negative Big, respectively. During the fuzzification process, the membership degree of each variable belonging to each fuzzy set is calculated based on the membership functions.

To meet the accuracy and stability requirements of the longitudinal control algorithm for the time-varying nonlinear system of the intelligent vehicle, and to facilitate the characteristics and debugging needs in the subsequent simulation stage, the domains of the input variables *e*_xv_ and *e*_c2_ in the longitudinal fuzzy control part are both set to [−10, 10]. The domains of the output variables *K*_p2_, *K*_i2_, and *K*_d2_ are set to [0, 1], [0, 1], and [0, 1], respectively.

In longitudinal fuzzy PID control, the inputs to the fuzzy controller are the weighted errors of longitudinal position and velocity, denoted as *e*_xv_, and the error transformation rate *e*_c2_. The outputs are the three parameters of the PID controller: *K*_p2_, *K*_i2_, and *K*_d2_. The intelligent vehicle adjusts the torque applied to the wheels, represented by *T*, to minimize both the longitudinal displacement error *e_x_* and the velocity error *e*_v_, driving them toward zero.

In each stage of longitudinal control, the strength of the proportional, integral, and derivative actions in the traditional PID control is determined based on the longitudinal weighted errors *e*_xv_ and its transformation rates *e*_c2_. The construction of the fuzzy rule table in longitudinal fuzzy control requires comprehensive consideration of the dynamic errors and overshoot in the longitudinal motion, excessive overshoot can impact ride comfort.

When the absolute value of the longitudinal weighted error *e*_xv_ is large, to expedite the reduction in deviation and enable the intelligent vehicle to quickly reach the longitudinal reference position and velocity, it is advisable to increase the adjustment magnitude, specifically by increasing the value of *K*_p2_. However, to prevent excessive overshoot from adversely affecting the stability of the vehicle’s operation and the comfort of the ride, the increase in *K*_p2_ should be determined based on the magnitude of *e*_c2_. Additionally, *K*_d2_ should be reduced to some extent to avoid premature braking, which could prolong the overall adjustment time.

When the absolute value of the longitudinal weighted error *e*_xv_ is large, in order to minimize the steady-state error of the control system as much as possible and reach the longitudinal position and velocity of the reference trajectory as quickly as possible, *K*_i2_ should be increased appropriately based on the magnitude of *e*_c2_. Additionally, the magnitude of *K*_p2_ should be controlled based on the result of whether the output oscillates. Meanwhile, the value of *K*_d2_ should also be increased appropriately. This approach can not only accelerate the rate of descent of the overshoot of the system and suppress it but also suppress the excessive control intensity caused by the previous large *K*_p2_, thus better ensuring the comfort of the intelligent vehicle’s motion.

When the absolute value of the longitudinal weighted error *e*_xv_ is small, indicating that the actual longitudinal position and velocity of the intelligent vehicle are close to the reference values, *K*_p2_ and *K*_i2_ should be kept relatively stable, while *K*_d2_ should be increased appropriately. This further avoids the generation of a certain degree of overshoot and steady-state error, ensuring the stability of the intelligent vehicle during its operation.

When the absolute values of both the longitudinal weighted error *e*_xv_ and its rate of change *e*_c2_ tend to zero, indicating that the intelligent vehicle is in a phase of constant-speed travel, to avoid excessive overshoot and maintain steady-state error within an acceptable range, *K*_d2_ should be increased appropriately, while *K*_p2_ and *K*_i2_ should be decreased. This ensures a more stable longitudinal control.

In summary, the longitudinal fuzzy PID controller should dynamically adjust the values of *K*_p2_, *K*_i2_, and *K*_d2_ based on varying conditions under different longitudinal weighted errors *e*_xv_ and their rates of change *e*_c2_, to ensure that the vehicle can smoothly, quickly, and comfortably control acceleration and deceleration to reach the reference position and speed within a short time. For large position and velocity deviations, it is advisable to moderately increase *K*_p2_, *K*_i2_, and *K*_d2_ values, within a relatively larger control range to promptly reduce the weighted error, while avoiding excessive overshoot. For a smaller position and velocity deviations, maintaining moderate *K*_p2_, *K*_i2_, and *K*_d2_ values while slightly increasing *K*_d2_, helps minimize steady-state error and ensure smooth control. When the longitudinal weighted error and its rate of change approach zero, allowing for a certain level of steady-state error, it is recommended to slightly decrease *K*_p2_, *K*_i2_, and *K*_d2_ values and moderately increase *K*_d2_ to ensure system stability and passenger comfort.

Based on the above analysis and adjustments made in subsequent chapters the following multiple simulations and experimental results, the fuzzy rule table for the longitudinal fuzzy PID controller is formulated, as shown in Table 2.

**Figure 9:**
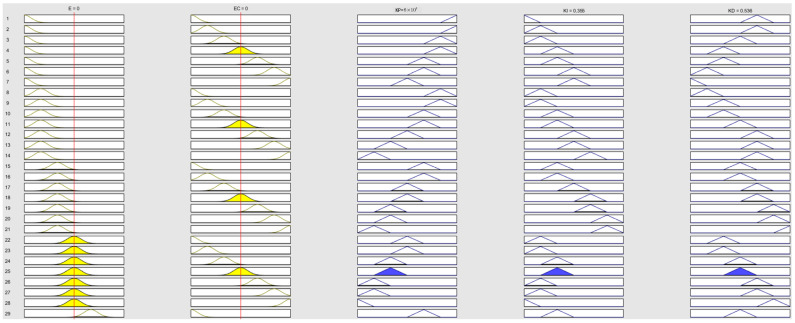
Longitudinal dynamic simulation results.

**Figure 10:**
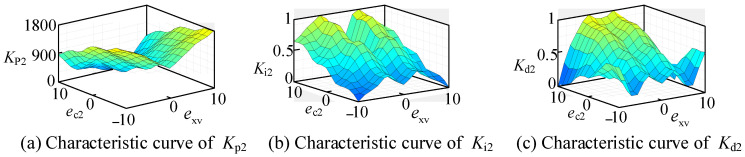
Longitudinal characteristic curves of input and output.

Using the rules described above, we input them into the MATLAB Fuzzy Logic Toolbox for dynamic simulation, with the results shown in Figure 9. Additionally, the characteristic curves of the input and output variables are shown in Figure 10.

## 4. Experimental Validation

**Figure 11:**
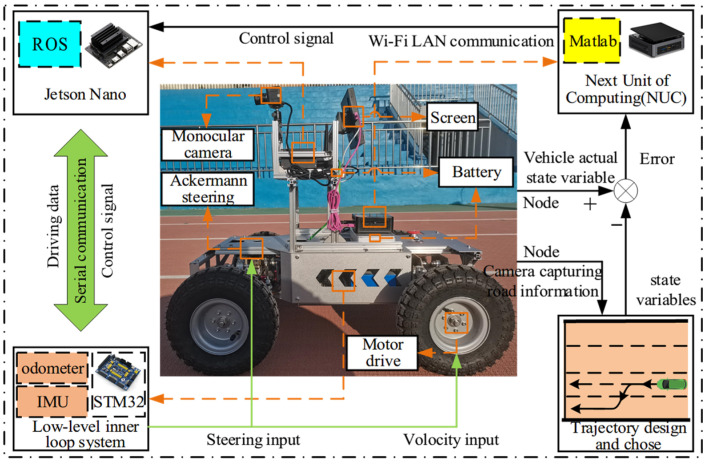
Experiment Configuration.

To validate the proposed control algorithm, experiments were conducted using an intelligent car with front-wheel Ackermann steering and rear-wheel motor drive, powered by batteries connected to screens, a Jetson Nano, and NUC (See Figure 11). The intelligent car’s subsystems are modularized, with lateral and longitudinal inputs represented by the front-wheel steering angle $\delta_{f}$ and longitudinal velocity *v*_x_, respectively. Sideways slippage is not considered during the experiments. The odometer provides the actual longitudinal speed *v*, lateral displacement *y*, and longitudinal displacement *x* to the longitudinal system, while the IMU provides the actual yaw angle θ to the lateral system.

The longitudinal fuzzy PID controller receives the weighted errors of longitudinal displacement and velocity *e*_xv_ as inputs and outputs the longitudinal velocity *v*_x_. The STM32 module communicates with Jetson Nano via serial port to transfer the actual state of the car, operating on the ROS platform, the Jetson Nano formats the actual state of the car and road information collected by the camera into multiple node files, which are then transmitted to Matlab/Simulink via WIFI LAN. In this process, the car’s actual state is sent to the controller, while the road information is used in the trajectory design and selection process. Additionally, the output control signals of the lateral and longitudinal fuzzy PID controllers can be also transmitted in the same way, enabling seamless information sharing among all components.

### 4.1. Operating Condition 1: Constant Speed Lane Changing and Straight Lane Driving

In this operating condition, the intelligent vehicle performs a lane change at a constant speed of 0.5 m/s for 40 s, followed by 10 s of straight-line driving, resulting in a total longitudinal displacement of $X_{1}\left(t_{f}\right)=25\;m$.

After debugging, the weights for lateral displacement error and yaw angle error are set to $\lambda_{1}=1$ and $\lambda_{2}=1$, respectively, while the weights for longitudinal displacement error and velocity error are set to $\omega_{1}=1$ and $\omega_{2}=0.1$, respectively. In the fuzzy control component of the lateral PID controller, the domains of the input variables *e*_yφ_ and *e*_c1_ are both set to [−3, 3], and the domains of the output variables *K*_p1_, *K*_i1_, and *K*_d1_ are set to [0, 34], [0, 7], and [0, 0.1], respectively.

**Figure 12:**
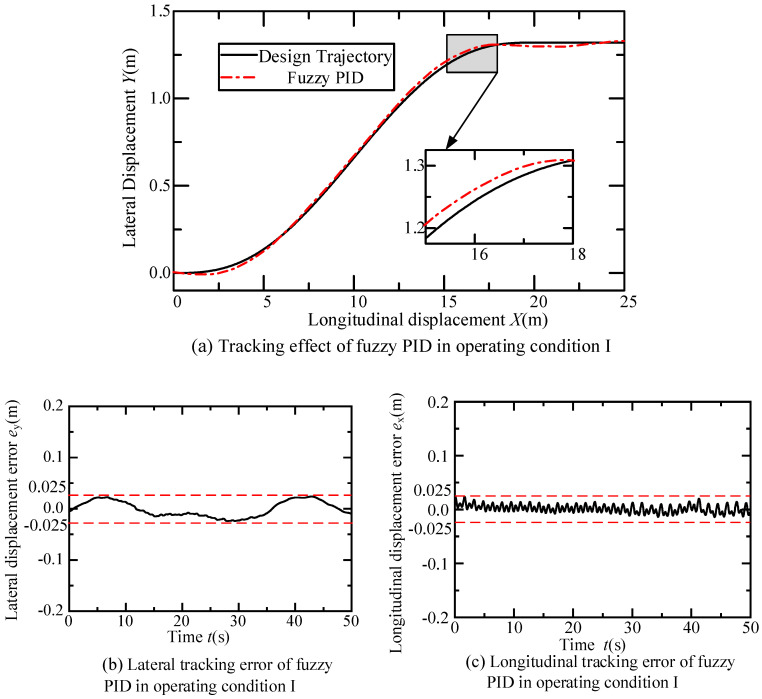
Diagram of the effect of the intelligent vehicle’s tracking trajectory in constant speed lane changing and straight driving experiments.

In the fuzzy control part of the longitudinal PID controller, the domains of the input variables *e*_xv_ and *e*_c2_ are both set to [−10, 10], and the domains of the output variables *K*_p2_, *K*_i2_, and *K*_d2_ are set to [0, 6], [0, 1], and [0, 2], respectively. The sampling time during the experiment is 0.05 s. The trajectory tracking performance of the fuzzy PID controller under this operating condition is shown in Figure 12.

**Figure 13:**
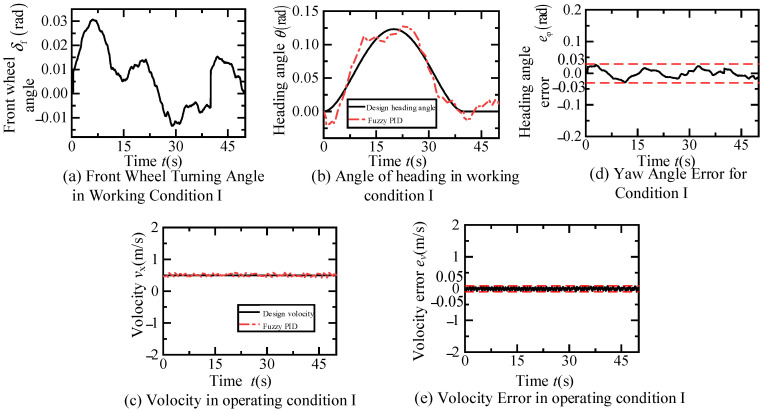
Time dependent variation in control variables and related errors in constant speed lane changing and straight driving experiments.

According to Figure 12a, the dual fuzzy PID controller effectively tracks the trajectory designed in scenario one. The vehicle first undergoes a 40 s lane change at a constant speed of 0.5 m/s, with a lateral distance of 1.32 m measured by the visual system, and a longitudinal distance set to 20 m. Subsequently, it travels straight at 0.5 m/s for 10 s. The inset of Figure 12a shows that as the lane change is about to end, the trajectory error gradually approaches zero. Furthermore, Figure 12b,c indicates that the lateral and longitudinal displacement errors during the scenario one experiment are both within 0.025 m. Finally, the variations over time of the front wheel steering angle, yaw angle and error, speed and error of the fuzzy PID controller in this scenario are illustrated in Figure 13. Figure 13a demonstrates the change curve of the front wheel steering angle change closely matches the required steering angle for the trajectory in scenario one. Figure 13b,d shows that the yaw angle of the fuzzy PID controller basically tracks the designed yaw angle during the experiment, with errors controlled within 0.03 radians. As depicted in Figure 13c,e, the fuzzy PID controller maintains the intelligent vehicle speed at approximated 0.5 m/s, with errors controlled within 0.05 m/s.

### 4.2. Scenario Two: Intersection Straight-Lane Parking

**Figure 14:**
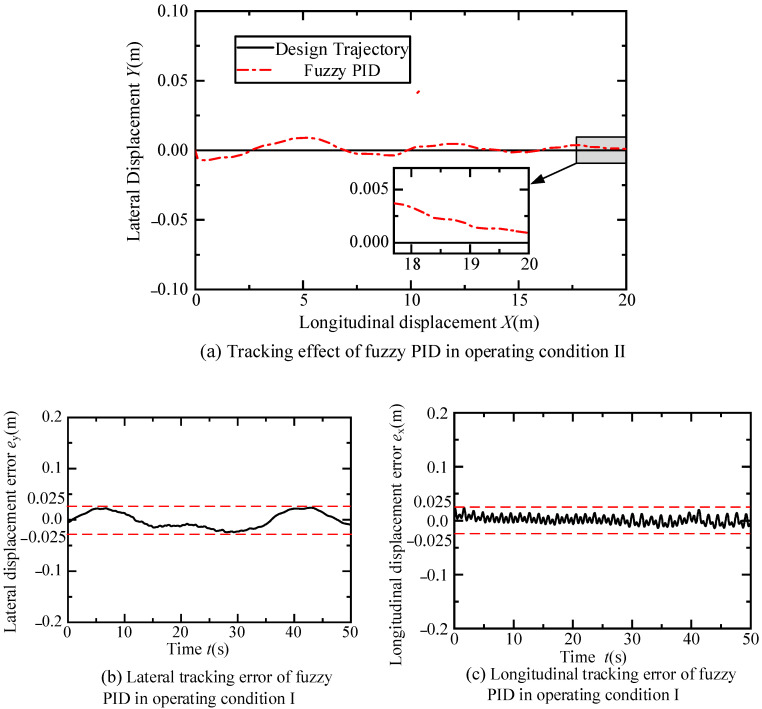
Diagram of the effect of the intelligent vehicle’s tracking trajectory in straight parking experiment at intersection.

To further validate the universality of the controller under two scenarios, the trajectory tracking performance of the fuzzy PID controller for stop-line parking at the intersection is demonstrated in Figure 14a.

As indicated, the dual fuzzy PID controller is capable of effectively tracking the trajectory designed in Scenario Two, where the vehicle decelerates to park at a speed of 0.5 m/s for 70 s with a longitudinal distance set to 20 m. It can be observed from the inset of Figure 14a that, in the final stage of the trajectory, the error gradually converges to zero. Furthermore, from Figure 14b,c, it is evident that during the experiment, the fuzzy PID controller controls the lateral displacement error within 0.01 m and the longitudinal displacement error within 0.05, and as the trajectory approaches its end, both lateral and longitudinal displacement errors tend towards zero.

**Figure 15:**
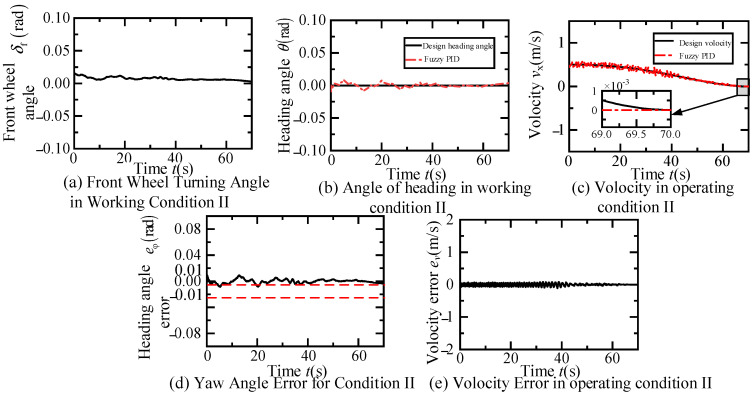
Time dependent variation in control variables and related errors in straight parking experiments at intersections.

Finally, the relationship between the front wheel steering angle, yaw angle and error, speed and error of the fuzzy PID controller under this scenario is illustrated in Figure 15. According to Figure 15a, it can be observed that the change in front wheel steering angle during the experiment closely aligns with the required steering angle for the motion trajectory of intersection deceleration parking in Scenario Two. As inferred from Figure 15b,d, the yaw angle of the fuzzy PID controller basically tracks the designed yaw angle during the experiment, with errors controlled within 0.01 radians. As shown in Figure 15c, the fuzzy PID controller effectively controls the vehicle’s speed to follow the speed curve designed in Scenario Two, and combined with the inset of Figure 15c,e, it can be observed that the speed error gradually converges to zero.

In summary, under the same controller parameters as in Scenario One, the dual fuzzy PID controller exhibits good lateral and longitudinal tracking effects in the intersection parking motion of Scenario Two. Additionally, all state variables of the intelligent vehicle remain within small and acceptable ranges.

## 5. Conclusions

In this paper, a two-wheel, two-degree-of-freedom vehicle dynamics model is established and transformed into the Frenet coordinate system to derive lateral and longitudinal tracking error models. Controllers are constructed for both lateral and longitudinal directions using fuzzy PID control. Fuzzy control is utilized to update the three PID parameters in real time based on the sampling time to enhance control performance. Finally, a joint intelligent vehicle experiment is conducted for validation.

The trajectory design and control systems are meticulously simulated within a virtual environment, while actual vehicle experiments are conducted on a simulated road to validate the systems’ performance and reliability. The setup uses STM32 as the inner loop module, Jetson Nano as the upper controller, and NUC as the PC end. Communication between the three parts is achieved via serial port communication and WIFI LAN communication for efficient information sharing. In addition, ROS and Simulink serve as software platforms, enabling the publication and subscription of road condition, IMU, and odometer data in a node-based manner. An intelligent vehicle joint experiment platform equipped with a monocular camera is established to validate the accuracy, rationality, and effectiveness of visual detection and recognition, trajectory design under different scenarios, and the dual fuzzy PID controllers for both lateral and longitudinal directions.

## Figures and Tables

**Table 1:** Fuzzy rules in horizontal fuzzy PID controller.

*e*_yφ_ */e*_c1_	NB	NM	NS	ZO	PS	PM	PB
	*K*_p1_, *K*_i1_, *K*_d1_						
NB	PB NB PS	PB NB PS	PM NB ZO	PM NM ZO	PS NM ZO	PS ZO PB	ZO ZO PB
NM	PB NB NS	PB NB NS	PM NB ZO	PM NM NS	PS NS ZO	ZO ZO PS	ZO ZO PM
NS	PM NM NB	PM NM NB	PM NS NM	PS NS NS	ZO ZO ZO	NS PS PS	NM PS PM
ZO	PM NM NB	PS NM NB	PS NS NM	ZO ZO NS	NS PS ZO	NM PS PS	NM PM PM
PS	PS NS NB	PS NS NM	ZO ZO NS	NS PS NS	NS PS ZO	NM PM PS	NM PM PS
PM	ZO ZO NM	ZO ZO NS	NS PS NS	NM PM NS	NM PM ZO	NM PB PS	NB PB PS
PB	ZO NB PS	NS NM ZO	NS NS ZO	NM ZO ZO	NM PS ZO	NB PM PB	NB PB PB

**Table 2:** Fuzzy rules in longitudinal fuzzy PID controller.

*e*_xv_/*e*_c2_	NB	NM	NS	ZO	PS	PM	PB
	*K*_p2_, *K*_i2_, *K*_d2_						
NB	PB NB PS	PB NM PS	PM NM ZO	PM NS ZS	PS NS NS	PS ZO NM	ZO ZO NB
NM	PM NM NM	PM NM NS	PS NS ZS	PS NS ZO	ZO ZO PS	NS ZO PS	NM PS PS
NS	PS NS ZO	PS NS ZO	ZO ZO PS	ZO PS PS	NS PS PM	NS PM PM	NM PM PB
ZO	ZO NM NS	ZO NM NS	NS NS ZO	NS NS ZO	NM NM PS	NM NM PS	NB NB PM
PS	PS NS ZO	PS NS ZO	ZO ZO PS	ZO PS PS	NS PS PM	NM PM PM	NB PM PB
PM	PM NM NM	PM NM NS	PS NS NS	PS NS ZO	ZO ZO PS	NS ZO PS	NM PS PS
PB	PB NB PS	PB NM PS	PM NM ZO	PM NS NB	PS NS NB	PS ZO NM	ZO ZO PS

## Data Availability

The datasets presented in this article are not readily available due to privacy restrictions.
